# A Young Boy with 21q21.1 Microdeletion Showing Speech Delay, Spastic Diplegia, and MRI Abnormalities: Original Case Report

**DOI:** 10.1055/s-0043-1774291

**Published:** 2023-08-31

**Authors:** Piero Pavone, Raffaele Falsaperla, Martino Ruggieri, Simona Domenica Marino, Enrico Parano, Xena Giada Pappalardo

**Affiliations:** 1 Department of Child and Experimental Medicine, Section of Paediatrics and Child Neuropsychiatry, University of Catania, Italy; 2 Unit of Pediatrics and Pediatric Emergency, University Hospital Policlinico “G. Rodolico-San Marco,” Catania, Italy; 3 Neonatal Intensive Care Unit, San Marco Hospital, University Hospital Policlinico “G. Rodolico-San Marco,” Catania, Italy; 4 National Council of Research, Institute for Biomedical Research and Innovation (IRIB), Unit of Catania, Italy

**Keywords:** 21q21.1 deletion, speech delay, brain anomalies, microRNA, copy number variant

## Abstract

Chromosome 21q deletion syndrome is a rare disorder affecting the long arm of chromosome 21 and manifesting with wide phenotypic features depending on the size and position of the deleted region. In the syndrome, three distinct deleted regions have been distinguished: region 1, from the centromere to approximately 31.2 Mb (21q11.2-q22.11); region 2, from 31.2 to 36 Mb (21q22.11-q22.12); and region 3, from 36 to 37.5 Mb to the telomere (21q22.12-q22.3). The clinical features are highly variable manifesting with mild, poorly recognizable signs or with severe symptoms including craniofacial dysmorphism, growth failure, developmental delay, behavioral/affective abnormalities, and systemic malformations. We report here the case of a young boy with speech delay, mild spastic diplegia, and brain anomalies on magnetic resonance imaging (MRI). The genetic analysis displayed a microdeletion of the long arm of chromosome 21 approximately extending up to 1.08 Mb. Clinical presentation of the patient and cases of 21q21 deletion reported by the literature are discussed.

## Introduction

Chromosome 21q deletion syndrome (ORPHA574) is a very rare genetic disorder caused by missing of the genetic material in the long arm of chromosome 21. 1 2 3 Similarly to other chromosome anomalies, the syndrome may present with different clinical features depending on the size and position of the deleted region. 4 5 6 The clinical spectrum is highly variable, ranging from irrelevant clinical impairments to severe phenotypic features including craniofacial anomalies, intellectual disability, autistic spectrum disorder (ASD), brain malformations, and systemic disorders. 7 8 9 In this syndrome, three distinct deleted regions have been described: region 1 ranges from the centromere to approximately 31.2 Mb (21q11.2-q22.11, containing about 50 genes); region 2 from 31.2 to 36 Mb (21q22.11-q22.12, containing about 80 genes); and the region 3 from 36 to 37.5 Mb to the telomere (21q22.12-q22.3, containing more than 130 genes). 2 6

Errichiello et al 7 proposed distinguishing the 21q deletion of region 1 in two subregions: subregion 1, a segment from the centromere to approximately 21 Mb (21q21.1), and subregion 2, with a length of almost 32 Mb (21q22.11). They maintain that patients with subregion 1 deletion are associated with intellectual disability, whereas patients with subregion 2 deletion are affected by neurobehavioral disorders including obsessive-compulsive disorder, poor social interactions, and vulnerability to psychosis. Basically, deletions in regions 1 and 2 usually involve a more severe phenotype, whereas deletions in region 3 show a mild clinical phenotype. 1 6

Here, we report the case of a 3-year-old boy with a microdeletion extended to approximately 1.08 Mb involving the 21q21.1 region. This copy number variant (CNV) belongs to region 1 and subregion 1 according to the above classification 2 6 7 and, to our knowledge, it also represents the smallest deletion reported in the literature. The clinical manifestations presented by the boy were not severe and consisted mainly of congenital symmetric plagiocephaly, speech delay, and spastic diplegia of mild type with brain magnetic resonance imaging (MRI) anomalies consisting of multiple areoles of altered signals in frontoparietal subcortical white matter area.

## Clinical Findings

**Fig. 1:**
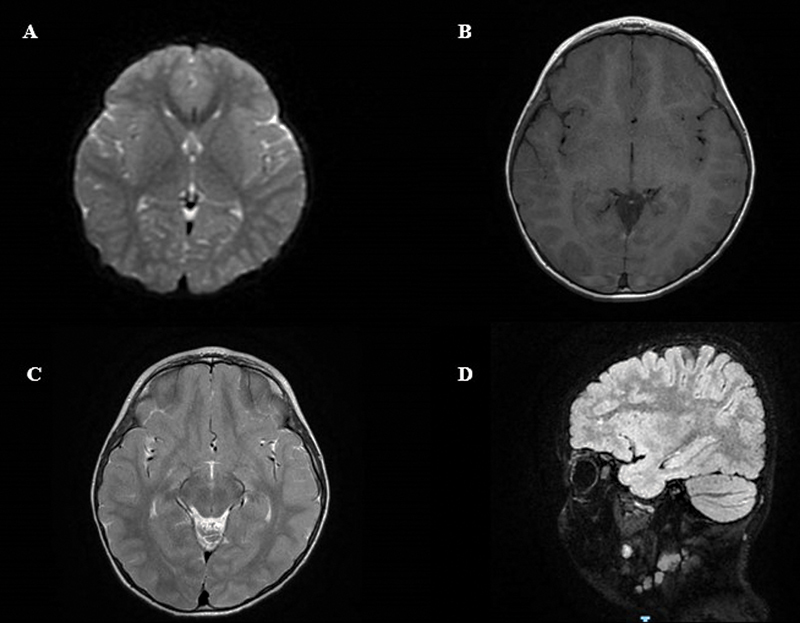
( **A** – **D** ) Magnetic resonance imaging (MRI) of the brain anomalies of the proband. Brain MRI showed nondilated fourth ventricle in axis, regular triventricular and supratentorial systems, and corpus callosum normal for morphology and signals. Multiple small areas of altered signals characterized by hyperintensity of signals were found in the sequences with long repetition time (TR) with prevalent perivenular distribution within the subcortical white matter in the frontoparietal area bilaterally (more numerous in the frontal site).

This 3-year-old boy is the second born of unrelated Italian parents. His 5-year-old brother is healthy. Family history is irrelevant. The parents are healthy with a good level of instruction. At the time of conception, the age of the father was 31 years and the mother was 29 years old. The mother referred having normal fetal movements without complications and infectious illnesses during the gestation period. Intrauterine ultrasound did not show fetal anomalies. The child was born at full term with normal delivery. The Apgar scores were 8 at 1 minute and 10 at 5 minutes. The birth weight was 2.900 kg, height was 49 cm, and occipitofrontal circumference (OFC) was 34 cm, all within normal limits. At birth, no dysmorphic features were noted with the exception of a symmetrical plagiocephaly. The first months of life were uneventful, but around 16 to 18 months, a delay in the stages of psychomotor developmental was noticed. At 21 months, he was first admitted to the Pediatric Section, San Marco-Polyclinic Hospital (Catania, Italy), due to severe speech delay and tiptoe walking. At physical examination, the child appeared in good condition. A marked symmetrical plagiocephaly was noted. Heart, thorax, abdomen, and genital organs were normal. At the neurological examination, the child was alert and the cranial nerves were grossly intact. However, muscle hypertonia of the inferior legs with tiptoe walking and patellar tendon reflexes tapped briskly were observed. The child showed a significant speech delay, since he was able to use only a single word, which was not easily intelligible. Pupils were isochoric, isocyclic, and normoreactive to light. At routine laboratory analysis, electrolytes, plasma and urinary amino acids, thyroid markers, organic acids, plasma purine, and total cholesterol were within normal limits. Fundus examination and hearing exploration were normal, so were electrocardiogram (ECG) and ECG and electroencephalogram (EEG) performed while awake and during sleep. Brain MRI did not show dilation of the fourth ventricle, regular triventricular and supratentorial systems, and normal corpus callosum for morphology and signals. Multiple small areas of altered signals characterized by hyperintensity of signals were found in the sequences with long repetition time (TR) with prevalent perivenular distribution within the subcortical white matter in the frontoparietal area bilaterally (more numerous in the frontal site; Fig. 1 ). The small area anomalies were also observed at the level of the corona radiata. No altered hypersignals at the cerebellar parenchyma and brainstem were reported with regular wideness of the cerebral sulci and cisterns. After a week, the child was dismissed with the diagnosis of congenital symmetrical plagiocephaly, speech delay, and mild spastic diplegia. Logotherapeutic treatment and physical therapy were started. At the last examination at 3.5 years, the child showed a weight of 15.5 kg, height of 97 cm, and OFC of 50.5 cm, all within normal limits. Cognitive evaluation indicated a normal development with a marked language delay as the child was able to pronounce only a few words. Despite his hyperactivity, he showed positive social interactions.

## Genetic Analysis

Genomic deoxyribonucleic acid (DNA) extraction was isolated from peripheral blood of the proband and parents to perform array-based comparative genomic hybridization (aCGH) using the SurePrint G3 Oligo ISCA 8 × 60k (v.2.0) platform according to the manufacturer's recommendation (Agilent Technologies Santa Clara, CA, United States). The microarray includes 60,000 oligonucleotide probes in which the average spacing between the probes is 60 kb with a resolution of 150 to 200 kb. Data analysis was done using CytoGenomics v.4.0.3 software and ADM-2 algorithm, and genomic coordinates were evaluated according to GRCh37/hg19 and converted in GRCh38/hg38. For the CNV interpretations were used to query the Database of Genomic Variants (DGV; dgv.tcag.ca) and the tracks available in the University of California Santa Cruz (UCSC) database (genome.ucsc.edu/), such as DECIPHER web-based resource (decipher.Sanger.ac.uk), Clinical Genome Resource (clinicalgenome.org), and CNV Morbidity Map Developmental Delay. 10

## Results

**Fig. 2:**
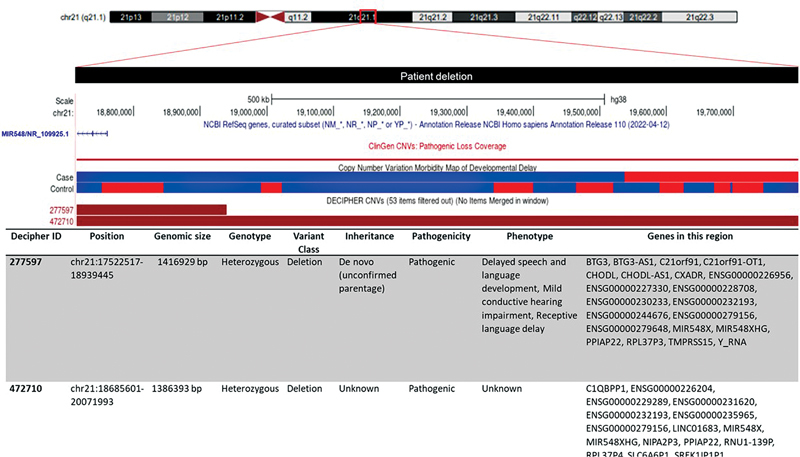
The genomic analysis of 21q21.1 microdeletion detected in the proband. The University of California Santa Cruz (UCSC) view shows the deleted region of our patient ( *black bar* ) overlapped with copy number variant (CNV) deletion entries of DECIPHER, ClinGen, and CNV morbidity databases. Entries are conventionally colored *red* for loss and *blue* for gain. The subtrack of ClinGen database shows that the highest number of losses among CNVs analyzed in this region are described as pathogenic variants. According to data of the CNV morbidity map of intellectual disability, the prevalent *blue-colored bar* indicates the accumulated level of gains compared to losses in cases and controls. For simplicity of the DECIPHER track view and for a more intuitive comparison, we filtered the DECIPHER patients based on the length of CNV (<2 Mb). Details of DECIPHER patient IDs (#277597 and #472710) are reported in the lower scheme.

The aCGH analysis of the patient revealed a microdeletion (1.8 Mb) of 21q21.1 (18,710,456–19,794,752) × 1 (hg38) containing a noncoding microRNA (miRNA) MIR548X (NR_109925.1). Results were found normal in the parent. Concerning the aCGH interpretation, data retrieved by the UCSC Genome Browser ( Fig. 2 ) indicated that the proband's CNV overlaps with other pathogenic deletions reported by the ClinGen database and DECIPHER patients (IDs: 277597 and 472710).

## Discussion

Chromosome 21q deletion syndrome is an uncommon genetic condition with a heterogeneous phenotype ranging from mild to severe clinical impairment. 7 Although it is well known that the severity of clinical manifestations depends on the size and position of the deleted region, the role of the involved genes and the specific genotype–phenotype correlation in several cases are yet to be established. The proband came to our observation due to the congenital symmetric plagiocephaly, speech delay, and tiptoe walking. Except for the congenital symmetric plagiocephaly, no additional malformities were observed. The child was alert with good social skills. The language was very poor as he was able to pronounce only few words, mostly not comprehensible. He also showed an increased muscle tone of the inferior legs with the tiptoe walking and a very active response to patellar tendon reflex. The brain MRI disclosed the presence of small multiple areas of hyperintense signals with TR with prevalent periventricular distribution located in the subcortical white matter bilaterally in the frontoparietal area and at the level of the corona radiata. The congenital symmetrical plagiocephaly suggested carrying out a genetic analysis. This CNV is located in region 1 and subregion 1 of 21q, according to the traditional model of 21q deletion classification by Lyle et al 2 and the more recent version proposed by Errichiello et al. 7

Case reports with 21q21.1 deletion are rarely described. Petit et al 11 studied three patients associated with a 7.9-Mb deletion in 21q21.1-21.2 (Decipher ID: #254181), and 3.8- and 8.5-Mb deletions in 21q21.1-21.3 (Decipher IDs: #276325; #274603). All of them were characterized by the loss of *NCAM2* (neural cell adhesion molecule 2) gene and affected by neurodevelopmental disorders, speech delay, and impaired social interaction. Another case (Decipher ID #231386) involving the *NCAM2* gene, with a 1.6-Mb deletion in 21q21.1-21.2, is reported by Scholz et al 12 who described a boy affected by ASD and macrocephaly. A relevant contribution on the study of the partial 21q monosomy is provided by Errichiello et al. 7 They report on five cases: three family members (father and two siblings) and two unrelated patients. The three family members (ClinVar IDs: #SCV000239859) carried a 10.6-Mb deletion mapping in 21q21.1-22.11, characterized by the loss of *GRIK1* (glutamate receptor, ionotropic kainate type subunit 1) gene. All of them showed mild clinical features including unspecific facial dysmorphism, tremor movements, behavioral disorder, and poor vocabulary. Patient 4 (ClinVar IDs #SCV000239860) showed a 14.5-Mb deletion in 21q11.2-21.3 encompassing the *RBM11* (RNA binding motif protein 11) and *BTG3* (B-cell antiproliferation factor 3) genes, associated with more severe clinical features compared with those observed in the three family members. Patient 5 (ClinVar IDs #SCV000239861) showed a 13.8-Mb deletion in 21q11.2-21.3 deletion and an additional deletion at 16p11.2, affected by severe clinical features including notable intellectual disability and a microadenoma at the center of hypophysis. The brain MRI showed enlarged cisterna magna in three patients and signals of ventricular white matter hyperintensity in two patients. 7 A retrospective analysis of seven pedigrees carrying 21q21.1-q21.2 aberrations was reported by Hu et al. 13 Among these seven pedigrees, a duplication in 21q21.11-q21.2 was found in six pedigrees and a deletion in 21q21.1-21.2 in one pedigree. Regarding the latter, the authors reported that all fetuses and family members showed normal phenotype. 13 Collectively analyzing the clinical data of aforementioned studies, 7 11 12 13 the results appear quite heterogeneous ranging from normal phenotype 13 to various clinical involvement including speech delay, global developmental delay, impaired social interaction, ASD, and movement disorder. 7 11 12 Discussing on the clinical features, we would emphasize a particular sign of this syndrome previously underrated.

In this study, tiptoe walking, as a sign of the mild spastic diplegia, was associated with brain MRI lesions consisting of multiple small areas of hypersignals with prevalent perivenular distribution located in the subcortical white matter mainly in the bilateral frontoparietal area. These cerebral anomalies may be suggestive of gliotic lesions, neurocellular patterns following various types of central nervous system injuries. Of note, the periventricular cerebral subcortical white matter hyperintensity found in the proband was also reported in two of the cases in Errichiello et al. 7 Reasonably, the cerebral abnormality might be potentially suggested to derive from the chromosomal defect interfering with the normal brain development process and causing the cerebral lesions. The 21q21.1-21.2 region contains several protein-coding and noncoding genes. 7 13 Among them, the most common genes associated with the chromosome 21q deletion syndrome are *NCAM2, GRIK1* , and *BTG3* . 7 11 12 The *NCAM2* gene has been pointed out in the three cases reported by Petit et al 11 and in the one case in Scholz et al. 12 This gene, belonging to the immunoglobulin superfamily, is known to play a role in neurodevelopment, in particular in controlling some neural-specific processes, such as neuronal differentiation, synaptogenesis, and memory formation. 14 Moreover, its impairment has been associated with cerebral malformations. 15 The *GRIK1* gene codes an ionotropic glutamate receptor of the kainate family involved in various neurophysiologic processes. *GRIK1* mutations have been reported in patients with behavioral disorders, schizophrenia, anxiety disorders, and epilepsy. 7 9 16

The *BTG3* gene is a member of the PC3/BTG/TOB family of growth inhibitory genes, expressed in several human tissues, alterations of which have been associated with intellectual disability. 7 Interestingly, in mice it has been shown to be highly expressed in the ventricular zone of the developing central nervous system. 13 17 18 19

In the present case, the deleted region contains the miRNA MIR548X (NR_109925.1). According to the miRDB database (mirdb.org), among the 2,418 predicted targets for MIR548X (also termed hsa-miR-548x-3p), the *BTG3* and *C21orf91* (chromosome 21 open reading frame 91) genes are included, as previously reported. 11 13 In particular, *C21orf91* seems to be involved in the susceptibility to herpes simplex labialis, 20 and also in the cerebral cortex neuron differentiation. 21

In the field of pediatric neurology, the involvement of miRNAs in brain development and neurodevelopmental disorders has been recently studied. 22 The loss or dysregulation caused by miRNAs has been associated with abnormal brain development. 23 24 The functional interaction between miRNA and its targets could be implicated in the disruption of some regulatory mechanisms of genome stability and coordination between transcriptional and posttranscriptional regulation of gene expression. Therefore, loss of MIR548X could potentially interrupt the functionality of several gene targets implicated in neural development. 23 We are aware of the limitations in our research, one of which is the lack of further molecular investigation (i.e., fluorescence in situ hybridization [FISH] assay) to confirm the deletion in the present patient. Mild clinical manifestations associated with genetic finding of microdeletion of 21q21.1 may be helpful to address future investigations in the diagnostic complexity of this syndrome.

## Conclusions

The present case corroborates the wide variability of clinical features of 21q21.1 deletion, which can be asymptomatic as in the case of Hu et al 13 or present with speech delay and spastic diplegia in mild form, as in our proband, in contrast with the cases described by Petit et al, 11 Errichiello et al, 7 and Scholtz et al, 12 in which neurological impairment was remarkable with signs of global developmental delay, impaired social interaction, and ASD. Of note, the presence of cerebral anomalies observed in the proband and in some cases of Errichiello et al 7 may be indications of lesions caused by 21q deletion during the process of neurodevelopment.
